# Ten simple rules for investigating (meta)genomic data from environmental ecosystems

**DOI:** 10.1371/journal.pcbi.1010675

**Published:** 2022-12-08

**Authors:** Paton Vuong, Michael J. Wise, Andrew S. Whiteley, Parwinder Kaur

**Affiliations:** 1 UWA School of Agriculture & Environment, University of Western Australia, Perth, Australia; 2 School of Physics, Mathematics and Computing, University of Western Australia, Perth, Australia; 3 The Marshall Centre of Infectious Diseases, School of Biological Sciences, The University of Western Australia, Perth, Australia; 4 Centre for Environment & Life Sciences, Commonwealth Scientific and Industrial Research Organisation (CSIRO), Floreat, Australia; Dassault Systemes BIOVIA, UNITED STATES

## Introduction

Metagenomics is the sequencing and study of DNA directly from environmental samples and is an approach used in microbial ecology to explore the diversity and functions of microbial communities [1]. The study of the metagenome presents an avenue to investigate environmental microorganisms in-situ through culture-independent techniques, which facilitates the discovery and analysis of the metabolic potential of hitherto uncultured taxa [2]. Expanding our reach into the once obscure facets of microbial life has yielded further insights into the ecological activity of microorganisms, uncovering a trove of novel metabolic products and pathways with a wide range of biotechnical applications in many industries [3,4]. Data obtained from metagenomes have also been pivotal in directing strategies for the cultivation of novel microorganisms [5]. The benefit of metagenomics is that it forms a wide-reaching preliminary approach that can provide contextual information to direct further downstream research for microorganisms in the wet lab.

Microbial ecology has developed to a stage where “big data” is often the norm. Through advances in sequencing and computational technologies, we have experienced an unprecedented level of progress in the field of microbial ecology, improving our understanding of the many global ecosystems [6]. Metagenomics draws many parallels when compared to data science—environmental sequence data presents a large volume of information that must be sifted through and sorted to make sense of underlying ecological and taxonomic patterns [7]. The amount of information that needs to be processed, however, can be daunting for those starting out and often still pose challenges even for those familiar to the field.

**Fig 1:**
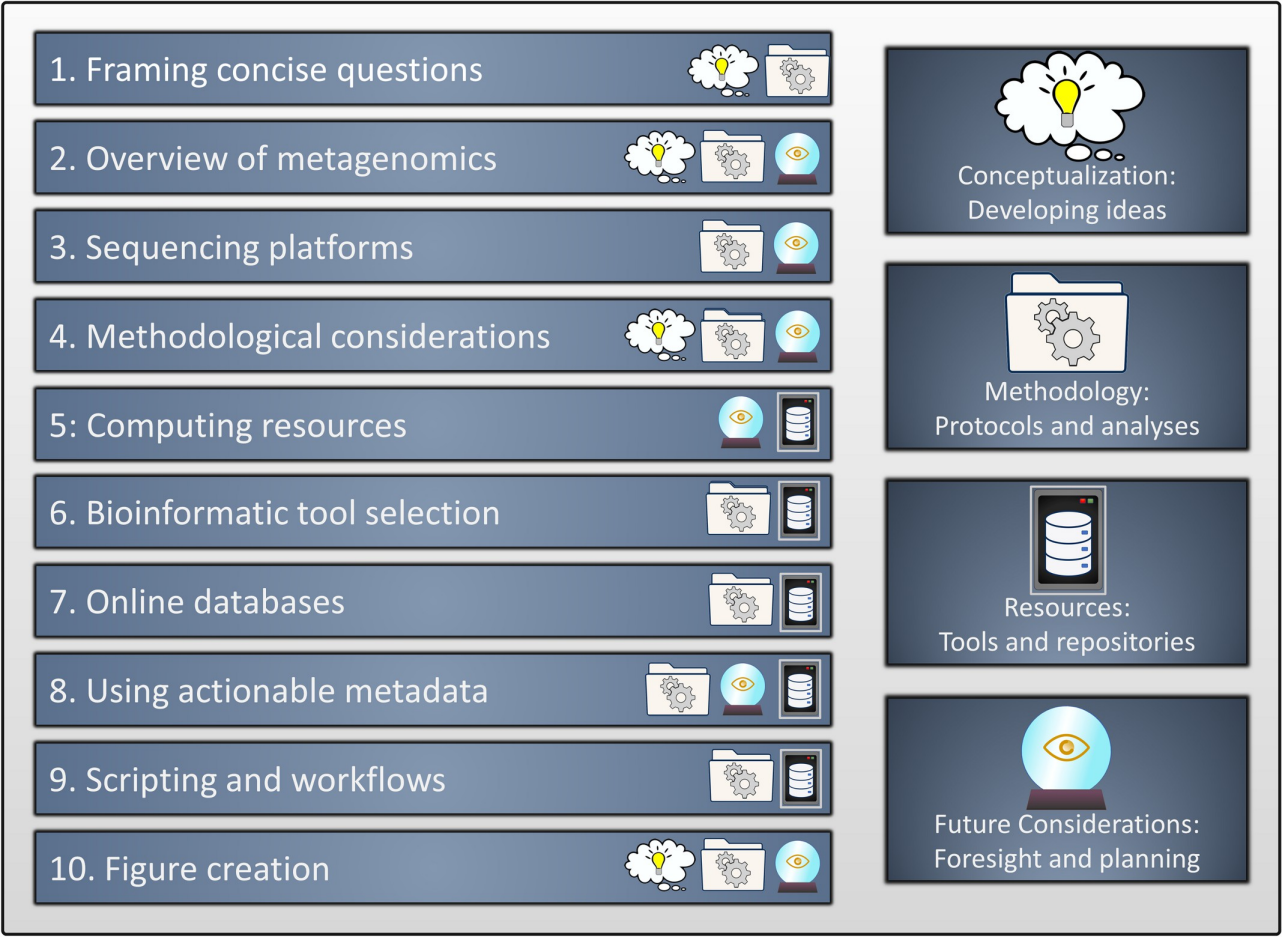
The 10 points outlining the metagenomic approach of microbial ecology. The summary of what is presented, along with the vital aspects discussed within each stage.

The inspiration behind this writing is from a recommendation to a PLOS Ten Simple Rules article on command-line bioinformatics [8]. The article provided a lot of information on tools and concepts used by bioinformaticians and it would have been a valuable source of guidance for many. This paper styled in the typical format of a Ten Simple Rules article, presents a guide on the basics of metagenomic-based research aimed at environmental settings. We will explore the concepts, tools, and resources utilized in metagenomics and how these can potentially help the progress of a researcher beginning their studies (Fig 1). The 10 simple rules format is a casual one and people are often busy, so at the end of each rule is a TL; DR (too long; didn’t read) that summarizes the point we are trying to make if you want to skim through (although we do hope you’ll stick around and read the paper in depth!).

## Rule 1: Plan a goal or you run the risk of an endless race

The drivers of microbial ecology are multifaceted, with almost limitless interactions and dimensions to explore, can cause a “paralysis of choice” scenario to occur. An example of this occurred during our first experience with data from a soil ecosystem. The learning process extremely captivating and through experimenting with a range of bioinformatic tools, we were able to produce an impressive amount of experimental data. However, generating data alone does not constitute a paper and that set of data produced ultimately ended up unused. What was lacking was a solid goal—one that could drive a clear question or a distinct narrative from the ecosystem of interest.

Metagenomics are preliminary studies that set the tone for further research, and one of its strengths is the ability to allow in-situ observation of both taxonomy and metabolic capacity within microbial populations within any given ecosystem. To find the “take home message” from your study, you need to ask yourself these questions regarding your research:

*What is the ecological aspect that you are showcasing*–This is arguably the most critical point as it forms the key focus of your study from which you create your narrative. Is it an ecosystem type, physicochemical properties, a group of taxa, or specific biosynthetic pathways that poses an interest to you?

*What question are you asking*–In order to produce a concise answer, you first need to pose a clear question. What insight are you trying to achieve from investigating the ecological aspect of interest?

*What has been done before*–Thorough consideration of current literature helps create a sort of “experimental control” that assists in validating the ecological observations obtained downstream. Knowing the current limits of understanding in the area of interest you plan to investigate means your study can better target the crucial knowledge gaps that need to be explored, while also providing a grounded premise with suitable comparison points.

*Are there notable differences/what has changed*–Are there any observed changes between your sample dataset or against known datasets? Is there a potential ecological driver that you can identify that could drive the observed differences?

*How does this compare to published literature*–Does your data reflect what is seen in previous literature? What insights can be gleamed from the differences/similarities within the expected results?

The major advantage of environmental metagenomics is that it is able to assess the functional potential of microbial communities and short-list candidate taxa for metabolic pathways of interest on a large scale. In the process of addressing your questions, the insights developed can be used to help guide the subject towards future studies. These could be pertinent aspects you wish to investigate further, or new perspectives that were unveiled from your studies (Box 1).

**Box 1. TL; DR–Rule 1**

Metagenomics is a vital preliminary driver for future research that allows you to delve into the almost limitless ecological relations within an ecosystem. Plan a concise narrative by focusing the question on a key taxonomic, functional, or physicochemical aspect, and ground your study against current literature that has explored your system of interest.

## Rule 2: Understanding the meta behind metagenomics

When searching for answers using metagenomics, it is important to understand what information the metagenome can provide and what the limitations are. Although metagenomics provides a tool for rapidly surveying microbial populations, the information obtained regarding the functional potential of the microorganisms therein is merely descriptive and not prescriptive. The presence of a gene may infer the potential existence of a metabolic function, but this does not guarantee that the associated microorganisms will produce the biological activity predicted [9]. Nonetheless, metagenomics presents a scalable avenue of exploration to infer microbial candidates and/or biological activities of interest, hence why its strengths lie as a preliminary study to help direct the scope of future research towards microbial life in various environmental systems.

Metagenomics as a form of molecular microbial ecology allows researchers to delve into the microbiome through the exploration of environmental DNA. The 2 most common approaches are marker-gene (also known as amplicon) sequencing, which uses PCR amplification to target select regions within genes of interest (commonly 16S rRNA for bacteria/archaea, internal transcribed spacer or ITS region for fungi, and 18S rRNA for eukaryotes), and whole-genome shotgun (WGS) sequencing that sequences all DNA present in a random but all-encompassing manner within the microbiome [10]. The main difference in the 2 sequencing approaches is that marker-gene sequencing is usually limited to providing taxonomic information only, whereas WGS sequencing provides both taxonomic and functional annotation, along with the potential to recover metagenome-assembled genomes (MAGs) [11]. Although contentious, usually only WGS-based studies are considered metagenomics as it explores the environmental genome in its entirety compared to the narrower scope of marker-gene approaches. Both however, have their place in molecular microbial ecology and are dependent on the extent of the questions posed by the study (from which hopefully Rule 1 has helped you define for yours).

For metagenomics using WGS data, there are 2 major approaches that can be taken: read-based (assembly free) or assembly-based analyses. A paper by Tamames and colleagues [12] explores the pros and cons of these 2 options and how they performed in regard to taxonomic and functional annotation using short-read sequences (more on this platform in Rule 3). Read-based approaches are quicker and retrieve more functions, but often results in the overprediction of functional genes. Read-based analyses are also extremely dependent on the quality of reference databases during homology searches, with a notable decrease in predictions when searching against less represented taxa. Assembly-based methods utilize longer sequences that provide advantages when classifying rare and distant homologies, but the assembly process itself is time consuming and requires more computational resources. The effectiveness of both approaches is also highly dependent on the microbial community structure present within the sampling environment, with high complexity microbiomes reducing the quality of assembly, thus favoring the use of read-based methods. Ultimately, the approach you chose depends on the target environment you wish to explore—small and easily characterized microbiomes are more amenable to read-based approaches, whereas less-studied niches with potentially novel taxa favor the use of assembly-based methods (Box 2).

**Box 2. TL; DR–Rule 2**

Metagenomic data is useful for inferring the taxonomy and functional potential of microorganisms on a large scale. By thoroughly defining your needs, you can better plan your approach on how you will explore the metagenome and obtain the appropriate data needed for your studies.

## Rule 3: The long and short of sequencing

With your question in hand, now comes the time to obtain the data from your environmental sample. The most common form of next-generation sequencing is short-read sequencing, which has enabled the ability to generate sequence data on a massive scale, providing the “big data” often seen in many metagenomic studies [13,14]. Short-read data was further enhanced through the development of sequence assembly software, which allowed for the construction of long contiguous sequences or “contigs” enabling the assembly-based analyses discussed in Rule 2. The information gleamed from assembled short-read sequences was further improved with binning software, which groups contigs through structural comparisons of their sequences, allowing for the recovery of MAGs. A major weakness of short-read assembly is that the shorter sequence lengths (usually between 200 to 550 base pairs) have issues covering certain genomic regions: long repeats, homologous regions between close strains, and sequences derived from low abundance species are some common issues that pose problems to the algorithm commonly used by short-read assemblers [13,15]. These issues lead to errors in assembly, leaving gaps in genomic data that can reduce the integrity of downstream analyses. However, the large volumes of data that can be provided at relatively low cost per base means that short-read sequencing is still a mainstay of many metagenomic studies.

Succeeding the short-read technologies is the aptly named long-read sequencing, with Oxford Nanopore Technologies (https://nanoporetech.com/) and Pacific Biosciences (https://www.pacb.com/) leading the forefront of this platform. The inclusion of these long-read sequencing platforms in recent metagenomic studies has shown to produce high-quality MAGs, which were able to resolve close lineages [16] as well as contain full length 16s RNA genes [17], both of which are difficult to produce when assembling using short-read sequencing alone. The longer length of sequences produced (average 30,000 to 50,000 base pairs, with record lengths exceeding 2.3 million) provides an advantage in metagenomic approaches by being able to resolve repetitive regions of DNA, with the added bonus of the longer sequences requiring fewer reads to assemble MAGs, thus reducing the chance of utilizing reads from errant species [14]. The main drawback of long-read platforms is they are more costly per base compared to short-read sequencing and require high-quality, high molecular weight DNA as input that can be challenging to obtain from complex environmental samples, and those with low biomass [18]. Care must be taken when considering DNA extraction protocols as many established commercial kits are usually designed with short-read sequencing in mind, such as kits that use beads to homogenize environmental substrates and lyse tough microbial cells, since the mechanical action of beat beating leads to excessive shearing of DNA.

The choice of sequencing platform depends on the questions you aim to ask in your study, as well as your available resources and budget. Short-read sequencing is the relatively cheap approach if all you need is bulk data from an environment and only aim to perform simple surveys of the microbial diversity. Being the predominant sequencing approach for the past decade, there is plenty of support for short-read sequencing in the form of established workflows and resources, allowing for an easier entry into metagenomics. For studies where the aim is a more comprehensive look at metabolic pathways or to recover candidate genomes, the inclusion of long-read sequencing will vastly increase the amount of information provided by coding sequences, improve the quality and recovery of MAGs, and provide better resolution to distinguish between sequences of highly similar strains. However, the improvements that long-read sequencing provides also requires more investments in time and resources in the form of specialized tools and customized protocols in order to reap the benefits of this added information (Box 3).

**Box 3. TL; DR–Rule 3**

The choice of sequencing platform depends on your experimental needs and the budget that you have access to for your study. Increasing the volume and/or length of the sequence data generated will also increase the required resources needed to properly prepare and process said data.

## Rule 4: Think outside the (computer) box

After deciding what sequencing platform to use, there are still steps that can be taken to improve the quality and recovery of data prior to performing computational work. The key aim in processing the “big data” in metagenomics is finding the hidden signals within the noise by processing raw sequence data into meaningful biological information. The in-situ modification of the microbiome through selective enrichment has shown to reduce community complexity and has aided in the recovery of rare microbial genomes [19]. Selective enrichment is also effective for targeting functions of interest by using nutrients that target the associated biochemical pathway(s), with a simplified microcosm shown to bring underrepresented taxa with relevant metabolic capabilities to the forefront [20]. Although it provides a straightforward approach, the enrichment method is not always applicable in all ecological studies, and there is no guarantee that the enriched members of the community provide meaningful roles in the overall bioprocesses that occur in the sample environment. Other techniques of elucidating rare taxa via in-situ methods include cell sorting, membrane diffusion, and microfluidic approaches but these can involve lengthy dilution processes and often require the successful isolation of the microbial candidates [5].

Methods that can help improve data recovery, especially in the binning of MAGs can also be applied during the sequencing step. High-throughput chromosome conformation capture, also known as “Hi-C,” is a technique that uses proximity ligation to link topographically related DNA fragments together [21]. A combination of WGS sequencing and paired-end sequencing of Hi-C sequences from the same sample can be used in tandem to improve quality of binned MAGs. Hybrid assembly approaches, which use long-read sequences as scaffolds for short-read sequences, have also been shown to improve the recovery and assembly statistics of MAGs from environmental samples when compared to the MAGs produced from the assembly of short or long reads alone [22]. Due to the ever-decreasing costs of sequencing, another way to improve data capture is to simply increase the sequencing volume. However, this brute force approach may not be a viable long-term solution as the advances in sequencing technology far outpace those of computational power, leading to the risk of generating more data than is able to be feasibly analyzed [23].

Metagenomic studies should not be limited to answering ecological questions but should also engage in considerations on how to improve the current methodology. Researchers should also take into account the techniques and strategies that can be applied even before any computational work occurs. After all, whatever occurs within the ecosystem and experimental protocols is ultimately reflected in the sequence data, and no amount of post-sequencing in-silico work can change this. Short of developing new bioinformatic tools, your experimental microcosm is arguably the place you have the most “creative” control, and more thought should be placed on how we can use this to our advantage to address common post-sequencing/computational limitations (Box 4).

**Box 4. TL; DR–Rule 4**

You should always aim to get the most of what you can out of your environmental/experimental sample(s). Methodological innovations are as important as ecological discoveries for furthering the field of molecular microbial ecology.

## Rule 5: Do not fear (super)computers. Fear the lack of them

After deciding your experimental protocols and obtaining your sequence data, you need to ensure you have the capacity to perform your computational analyses. In the information age and with the advent of “big data,” the field of data science requires heavy computational power to facilitate processing of large-scale data, and metagenomics is no exception to this rule. We quickly discovered that our run-of-the-mill laptop was in no way capable of handling the computational power needed for some of the data we needed to process. Without the help and high-performance computing services of the Pawsey Supercomputing Center (https://pawsey.org.au/), some of the larger sequenced metagenomes we worked with would never have been analyzed.

In 2020, the amount of raw sequence data generated by next-generation sequencing maintained by the INSDC exceeded 9 petabytes or over 9,000 terabytes [24]. As this data expands, classification software that utilizes these reference databases also need increasing computational resources to run. Examples include Kaiju [25], a taxonomic classification tool for metagenomic reads requires 118 to 133 GB to run using the NCBI nr database and similarly GTDB-tk [26], a tool used to classify prokaryotic genomes requires roughly 215 GB to classify the bacteria domain. Metagenomic assembly can require high amounts of RAM, with large and/or complex metagenomes such as those from soil requiring anywhere from 500 GB to over 1TB of RAM depending on the metagenomic assembler used [27]. In the words of Chief Brody from Jaws—“you’re gonna need a bigger boat.”

Nowadays, cloud computing and remote data storage are commonly used for genomic and other large-scale sequence analyses, and this recommended paper by Langmead and Nellore [28], lists a series of global cloud service providers and explains the concepts and strategies behind cloud computing. The previously mentioned “Ten simple rules for getting started with command-line bioinformatics” [8] also provides advice on estimating computational requirements and understanding your options when deciding on a computing platform. Nonetheless, your first port of call (for that aforementioned bigger boat) should be the providers that work with your institutions and their liaisons (Box 5).

**Box 5. TL; DR–Rule 5**

Big data needs equally big computers. Make sure you secure the computing resources you need for the work you intend to do.

## Rule 6: Metagenomic papers make for good window shopping for toolsets

A common bump in the road when starting research in metagenomics is during the selection of bioinformatic tools for processing data. This often results in a paralysis of choice as there are many available tools out there, but no agreed upon standard on how to implement them. Nowadays, there are many metagenomic workflow review papers that provide in depth explanations of all the typical avenues of analysis, along with suggestions for common bioinformatic tools at each step. A couple of papers we recommend that cover these aspects are by Pérez-Cobas and colleagues [10] and Bharti and Grimm [29], which not only provide detailed explanations behind the concepts of metagenomics in microbial ecology, but also present great flowchart figures on the processes for both WGS and amplicon sequence data analysis.

When deciding what to use when there are several options available, a good rule of thumb is to look at whether recent studies, within the last year or so, utilize those tools and importantly, if the tools themselves are regularly updated/maintained. Reviews for tools for a particular category (e.g., assembly, read alignment, binning) may also help in your decisions if you are feeling particularly stringent about a certain process. If the reviewed tools are prominent enough to be reviewed and are tested using Critical Assessment of Metagenome Interpretation (CAMI) methods, they can be considered solid recommendations. Try to ensure that the tools have undergone the CAMI second round challenge, which tests them against large and complex datasets, as well as those with long-read sequences, providing a more representative form of the current data observed within the field of metagenomics [30]. If all else fails, the best place to ask are other people in your research group—they can show you the ropes and can advise you on whether that tool is suitable for the task at hand (Box 6).

**Box 6. TL; DR–Rule 6**

Metagenomic papers make for good product reviews and evaluation. Stick to recent papers for relevant kit and ensure the tools you choose are still supported/updated.

## Rule 7: Databases are the key to everything

The essence of metagenomics as a data science is the data itself, with the sequence data and their subsequent derivatives such as genomes and reference gene/protein sequences being core resources in all metagenomic analyses. Whether you are sequencing your own dataset or using publicly available data, online databases are crucial repositories for the storing and sharing of large-scale sequence data. Traditionally, the most prominent online repositories for sequence data are those belonging to the International Nucleotide Sequence Database Collaboration (INSDC): the DNA Databank of Japan (DDBJ; http://www.ddbj.nig.ac.jp/), the European Molecular Biology Laboratory’s European Bioinformatics Institute (EMBL-EBI; http://www.ebi.ac.uk/ena/), and the National Center for Biotechnology Information (NCBI; https://www.ncbi.nlm.nih.gov/). INSDC organizations host a multitude of curated and annotated sequence data and is a valuable, publicly available resource for raw sequence data for exploration and reference data for classification.

Online databases are also crucial for the functioning of classification tools. Reference databases such as from RefSeq (https://www.ncbi.nlm.nih.gov/refseq/), Pfam (http://pfam.xfam.org/), and UniprotKB (https://www.uniprot.org/) form the basis for many search tools. The MIBiG 2.0 (https://mibig.secondarymetabolites.org/) and BiG-FAM database (https://bigfam.bioinformatics.nl/) are recently created online resources for the support of biosynthetic gene clusters (BGCs) mining tools. The Genome Taxonomy Database (https://gtdb.ecogenomic.org/) utilizes MAGs to expand the prokaryotic tree of life and is a vital resource for the classification of genomes recovered from environmental sequence data. Reference data is vital for identifying key taxa as well as their metabolic capacity, which is critical for making the connection between the core ecological drivers within the environmental setting.

Although reference datasets are a crucial part of database-driven analyses, users need to understand some of the limitations and shortfalls when relying on this data. The massive amounts of data generation means that when misclassification occurs, they can propagate rapidly even within nonredundant sequence databases [31]. A lack of consistent standards in database development and the lack of oversight in the submitted data, especially pertaining to reference genome completion and quality, are some of the major obstacles that affect the integrity of reference databases [32]. In addition, overrepresentation bias from sequence data obtained from abundant or more readily sequenced organisms can skew results in database-driven analyses. In 2022, NBCI proposed a remedy these biases through the creation of clustered databases, which both reduces the size of the total database and better represents the diversity of organisms and their associated sequences (https://ncbiinsights.ncbi.nlm.nih.gov/2022/05/02/clusterednr_1/). As discussed in Rule 2, users should be mindful when performing database-driven analyses on less-explored taxa that have few representatives in reference databases, which may cause novel candidates to be overlooked. Lastly, it is good practice to confirm your findings against a group of manually curated sequences obtained from well-characterized, closely related (or at least as close as possible) representative genes or genomes, to ensure that the classification provided by the large-scale datasets falls within the expected branch in the tree of life.

For data pertaining more to environmental studies, global sequencing initiatives such as the Earth Microbiome Project (https://earthmicrobiome.org/) and Tara Oceans (http://ocean-microbiome.embl.de/) provide publicly available sequence data that users can utilize and explore. Exploring shotgun metagenome data from Tara Oceans was one of the first forays on how to use bioinformatic tools during the candidature. This gave the ability to practice performing metagenomic analyses on real data collected from environmental samples. Accessible data provides a valuable resource for newcomers to metagenomics, and we highly encourage new users to grab a random metagenome to familiarize themselves on how to obtain and process sequence data for research (Box 7).

**Box 7. TL; DR–Rule 7**

There are plenty of repositories online from which you can find publicly available sequence data. These also provide key resources for classification and for (meta)genome mining, but users should be aware of their limitations and should check their findings against manually curated references.

## Rule 8: Context is (also) everything

Contextual information in metagenomics is crucial for discerning ecological patterns and relationships between the recovered sequence data and their physical environment. Metadata is the “data about the data” with its value increasing in importance as the collections of sequence data grow. The Genomic Standards Consortium (GSC; https://gensc.org/mixs/), an initiative for improving reporting standards in large-scale genomic data, recommended a series of standards for the “Minimum Information about any (x) Sequence” (MIxS), which includes genomes (MIGS), metagenomes (MIMS), and MAGs (MIMAG). Among the recommendations were the reporting of parameters such as physicochemical properties, spatial information, and geolocation, to help better understand the environment from where the sequence data was acquired. Contextual information is critical for filtering and categorizing the growing volumes of sequence data, allowing researchers to better understand the ecological drivers behind microbial communities and providing an avenue for developing better bioprospecting strategies towards microorganisms of interest.

Another important aspect of metadata is to ensure that the information is readily accessible and in an amenable form. The FAIR Principles (https://www.go-fair.org/fair-principles/) are a set of guidelines for improving the use and reuse of data in scholarly work, with a focus on the ability for machines to find and sort data with little human interaction. This allows for automated handling of the rapidly growing volumes data through scripting (which we will introduce in the next rule) and makes provisions for the data to be AI ready for machine-learning projects in the future. An example of actionable and amenable metadata can be found in the UniprotKB protein sequence database, which allows users to download associated metadata in different formats, such as tab-separated text or as an Excel spreadsheet, allowing the ability to parse and sort data quickly. Another example is the Integrated Microbial Genomes and Microbiomes system (IMG/M; https://img.jgi.doe.gov/m/), a genome and metagenome dataset repository with stringent metadata reporting protocols that follows the standards created by the GSC. Similarly, IMG/M provides easy access to sort and export metadata and has fields that allow users to filter by ecosystems and/or environmental factors. In one of our previous studies, we used the IMG/M database to find potential bioplastic producing candidates from their genome dataset and were able to utilize IMG/M’s searchable metadata to identify which environments were more likely to foster genotype candidates [33]. The study showed that metadata for large-scale datasets were a boon for bioprospecting strategies, as it allowed researchers to link target genotypes to their potential ecological distributions. Metadata is crucial to metagenomics and other similar fields of ecological study, and more efforts should be made to encourage better reporting and improving the actionability of contextual information across all sequence data platforms and repositories (Box 8).

**Box 8. TL; DR–Rule 8**

Contextual data is as valuable as the sequence data itself. Promoting actionable and accessible metadata is vital for the (re)use of the rapidly growing sequence data driving large-scale metagenomic studies.

## Rule 9: A little scripting goes a long way

The ability to write scripts is a valuable skill for a bioinformatician—not just for career prospects but also for your personal convenience when handling large volumes of data. Tasks such as running software on multiple files sequentially, making a pipeline to integrate a series of tools and their input and output (I/O) files, or parsing and/or organizing file data can be done with some simple scripts. Learning scripting languages can also help give a deeper understanding of the many bioinformatic tools available on the internet. A good resource for beginners wanting to learn code is Bionitio [34], a template system that teaches how to write command line tools for common languages found in bioinformatics (C, C++, Java, JavaScript, Perl, Python, R, and Ruby). The sample tool written for all languages in each Bionitio template reads FASTA files, generates statistics, and output the information in a tabular format (https://github.com/bionitio-team/bionitio). The format gives a baseline for the user to experience and understand code relevant to bioinformatics as well as help encourage good programming practices.

A programmer colleague of ours made the recommendation to be “language agnostic”—to not lock yourself into an exclusive language, but to be flexible in case the tools you need to use are spread across different scripting languages. However, as one can learn anything but not everything, here are some languages we can recommend as a starting point based on the many common software that we encountered when working with command-line-based tools. Foremost, Bash (https://www.gnu.org/software/bash/) is a must for command-line interactions as most terminals are Unix shells, and Bash is the primary way to explore and interact with files and folders. For more advanced data manipulation, we recommend Python as it is a relatively easy to learn language and has good readability. For learning Python, books we recommend are: “Practical Computing for Biologists” (https://practicalcomputing.org/) that is an all-encompassing guide on many aspects of software and computing practices common to in-silico microbial ecology and “Automate the Boring Stuff with Python” (https://automatetheboringstuff.com/) that teaches practical uses of the language, such as text searching and manipulating within files as well as how to produce various output formats, which is critical in creating pipelines and controlling file I/O actions. R (https://www.r-project.org/) is good to learn, not for scripting in the traditional sense, but for its strengths in statistical analysis and eminently for its ability to visualize data (more on that in the next section). RStudio (https://www.rstudio.com/) is an integrated development environment (basically a form of software to help you to write, use, and test code) for R used by beginner and advanced users alike and is highly recommended to be the first thing users should download after getting R. The best way to learn scripting is to get stuck into it and to utilize the many tutorials available online—once you get the basics down and grasp how data structures work the ability to automate processes will make your life much easier.

Another tip is to look how I/O files are used between bioinformatic tools and at the content contained within those files. As long as they are not in a compressed format, most I/O files used in bioinformatics are human readable through a text editor despite their seemingly odd or exotic file types. Understanding the I/O files can help you extract, filter, or organize content within files through scripts, as well as help set up efficient workflows through the use of workflow management systems like Snakemake (https://snakemake.readthedocs.io/) or Nextflow (https://www.nextflow.io/). Developing an efficient pipeline is a must due to the large volumes of data (and metadata) that are often present in metagenomics and workflow management systems can help transform your computational analyses into a scalable and reproducible format (Box 9).

**Box 9. TL; DR–Rule 9**

Learning to script helps to automate the processing of large-scale data and helps deal with other tedious, repetitive tasks. It can also help you understand the nuts and bolts behind how bioinformatic tools work and to help you develop efficient workflows.

## Rule 10: Make sure you have a great figure!

They often say a picture is worth a thousand words, which is handy as we can encapsulate the information from large-scale data by condensing it into a visual summary. A good figure should capture the attention of the reader and be a concise visual representation of your data. Fortunately, there is a multitude of tools that can help create the appropriate figure for the situation at hand. For phylogenetic trees, iTOL (https://itol.embl.de/) is a web-based platform that can create visually stunning trees with advanced annotation features. Circos (http://circos.ca/) is a software package that is great at creating circular visualization of data and is often used to create figures suitable for genomic data to present synteny between genomes. Some of best open-source graphics creation tools for use in data science are based in the R programming language, chief being the tidyverse package [35] containing a collection of R packages that create a variety of charts, all of which are demonstrated at the R Graph Gallery (https://www.r-graph-gallery.com/). R and its many packages are not just beneficial for graphical creations, but also for use in statistics, which can lend credence to both the tables and figures present in your research.

Image editing software is also a useful tool in figure creation allowing users to adjust headings, add extra details, as well as creating illustrations for conveying concepts and messages. GIMP (https://www.gimp.org/) and Canva (https://www.canva.com/) are free alternatives to commercial software for image editing. Journals will often require your figures to be high resolution (usually 300 to 400 dpi minimum) for clarity purposes. An alternative format to traditional image files is the Scalable Vector Graphics (SVG) that can be scaled in size without loss in quality, and many software packages, including those in R, allow graphs/plots to be exported as SVG format. Inkscape (https://inkscape.org/) is a free vector graphics editor designed for editing of SVG files, which then allows users to export their finished work in an image file format of their choice. The ability to create a good figure is crucial in getting the attention of your target audience and to communicate your findings and key messages of your research (Box 10).

**Box 10. TL; DR–Rule 10**

People like to look at pretty pictures! Use this fact to get people to pay attention to and understand your research through a good visual summary.

## Conclusion

The advent of next-generation sequencing and subsequent large-scale metagenomics has been responsible for the rapid advances in molecular microbial ecology, with further applications for use in in-silico bioprospecting. Analysis of environmental sequences has transformed this field of data science, which in turn has yielded successful contributions to both research knowledge and industrial applications from the novel discoveries within natural ecosystems. We hope that those who are looking to explore this field should endeavor not just to fill gaps in our ecological understanding of diverse microbial life, but also seek to develop innovations that address the ever-growing generation of environmental sequence data and develop strategies towards the many bottlenecks that come with the rapid increase of information.

## References

[1] Taş N, de Jong AEE, Li Y, Trubl G, Xue Y, Dove NC. Metagenomic tools in microbial ecology research. Curr Opin Biotechnol. 2021;67:184–191. doi: 10.1016/j.copbio.2021.01.019 33592536

[2] Latorre-Pérez A, Pascual J, Porcar M, Vilanova C. A lab in the field: applications of real-time, in situ metagenomic sequencing. Biol Methods Protoc. 2020;5(1):bpaa016. doi: 10.1093/biomethods/bpaa016 33134552 PMC7585387

[3] Madhavan A, Sindhu R, Parameswaran B, Sukumaran RK, Pandey A. Metagenome Analysis: a Powerful Tool for Enzyme Bioprospecting. Appl Biochem Biotechnol. 2017;183(2):636–651. doi: 10.1007/s12010-017-2568-3 28815469

[4] Marco DE, Abram F. Editorial: Using Genomics, Metagenomics and Other “Omics” to Assess Valuable Microbial Ecosystem Services and Novel Biotechnological Applications. Front Microbiol. 2019;10(151). 10.3389/fmicb.2019.00151 PMC6379446 30809205

[5] Lewis WH, Tahon G, Geesink P, Sousa DZ, Ettema TJG. Innovations to culturing the uncultured microbial majority. Nat Rev Microbiol. 2021;19(4):225–240. doi: 10.1038/s41579-020-00458-8 33093661

[6] Nayfach S, Roux S, Seshadri R, Udwary D, Varghese N, Schulz F, et al. A genomic catalog of Earth’s microbiomes. Nat Biotechnol. 2021;39(4):499–509. doi: 10.1038/s41587-020-0718-6 33169036 PMC8041624

[7] Navarro FCP, Mohsen H, Yan C, Li S, Gu M, Meyerson W, et al. Genomics and data science: an application within an umbrella. Genome Biol. 2019;20(1):109. doi: 10.1186/s13059-019-1724-1 31142351 PMC6540394

[8] Brandies PA, Hogg CJ. Ten simple rules for getting started with command-line bioinformatics. PLoS Comput Biol. 2021;17(2):e1008645. doi: 10.1371/journal.pcbi.1008645 33600404 PMC7891784

[9] Gutleben J, Chaib De Mares M, van Elsas JD, Smidt H, Overmann J, Sipkema D. The multi-omics promise in context: from sequence to microbial isolate. Crit Rev Microbiol. 2018;44(2):212–229. doi: 10.1080/1040841X.2017.1332003 28562180

[10] Pérez-Cobas AE, Gomez-Valero L, Buchrieser C. Metagenomic approaches in microbial ecology: an update on whole-genome and marker gene sequencing analyses. Microb Genom. 2020;6(8). doi: 10.1099/mgen.0.000409 32706331 PMC7641418

[11] Liu Y-X, Qin Y, Chen T, Lu M, Qian X, Guo X, et al. A practical guide to amplicon and metagenomic analysis of microbiome data. Protein Cell. 2021;12(5):315–330. doi: 10.1007/s13238-020-00724-8 32394199 PMC8106563

[12] Tamames J, Cobo-Simón M, Puente-Sánchez F. BMC Genomics. 2019;20(1):960. 31823721 10.1186/s12864-019-6289-6 PMC6902526

[13] Ayling M, Clark MD, Leggett RM. New approaches for metagenome assembly with short reads. Brief Bioinformatics. 2020;21(2):584–594. doi: 10.1093/bib/bbz020 30815668 PMC7299287

[14] Tedersoo L, Albertsen M, Anslan S, Callahan B. Perspectives and Benefits of High-Throughput Long-Read Sequencing in Microbial Ecology. Appl Environ Microbiol. 2021;87(17):e00626–e00621. doi: 10.1128/AEM.00626-21 34132589 PMC8357291

[15] Olson ND, Treangen TJ, Hill CM, Cepeda-Espinoza V, Ghurye J, Koren S, et al. Metagenomic assembly through the lens of validation: recent advances in assessing and improving the quality of genomes assembled from metagenomes. Brief Bioinformatics. 2019;20(4):1140–1150. doi: 10.1093/bib/bbx098 28968737 PMC6781575

[16] Bickhart DM, Kolmogorov M, Tseng E, Portik DM, Korobeynikov A, Tolstoganov I, et al. Generating lineage-resolved, complete metagenome-assembled genomes from complex microbial communities. Nat Biotechnol. 2022;40(5):711–719. doi: 10.1038/s41587-021-01130-z 34980911

[17] Singleton CM, Petriglieri F, Kristensen JM, Kirkegaard RH, Michaelsen TY, Andersen MH, et al. Connecting structure to function with the recovery of over 1000 high-quality metagenome-assembled genomes from activated sludge using long-read sequencing. Nat Commun. 2021;12(1):2009. doi: 10.1038/s41467-021-22203-2 33790294 PMC8012365

[18] Trigodet F, Lolans K, Fogarty E, Shaiber A, Morrison HG, Barreiro L, et al. High molecular weight DNA extraction strategies for long-read sequencing of complex metagenomes. Mol Ecol Resour. 2022;22(5):1786–1802. doi: 10.1111/1755-0998.13588 35068060 PMC9177515

[19] Delmont TO, Eren AM, Maccario L, Prestat E, Esen ÖC, Pelletier E, et al. Reconstructing rare soil microbial genomes using in situ enrichments and metagenomics. Front Microbiol. 2015;6(358). doi: 10.3389/fmicb.2015.00358 25983722 PMC4415585

[20] Naylor D, Fansler S, Brislawn C, Nelson William C, Hofmockel Kirsten S, Jansson Janet K, et al. Deconstructing the Soil Microbiome into Reduced-Complexity Functional Modules. MBio. 2020;11(4):e01349–e01320. doi: 10.1128/mBio.01349-20 32636252 PMC7343995

[21] Du Y, Sun F. HiCBin: binning metagenomic contigs and recovering metagenome-assembled genomes using Hi-C contact maps. Genome Biol. 2022;23(1):63. doi: 10.1186/s13059-022-02626-w 35227283 PMC8883645

[22] Overholt WA, Hölzer M, Geesink P, Diezel C, Marz M, Küsel K. Inclusion of Oxford Nanopore long reads improves all microbial and viral metagenome-assembled genomes from a complex aquifer system. Environ Microbiol. 2020;22(9):4000–4013. doi: 10.1111/1462-2920.15186 32761733

[23] November J. More than Moore’s Mores: Computers, Genomics, and the Embrace of Innovation. J Hist Biol. 2018;51(4):807–840. doi: 10.1007/s10739-018-9539-6 30140966

[24] Arita M, Karsch-Mizrachi I, Cochrane G. The international nucleotide sequence database collaboration. Nucleic Acids Res. 2021;49(D1):D121–D124. doi: 10.1093/nar/gkaa967 33166387 PMC7778961

[25] Menzel P, Ng KL, Krogh A. Fast and sensitive taxonomic classification for metagenomics with Kaiju. Nat Commun. 2016;7(1):11257. doi: 10.1038/ncomms11257 27071849 PMC4833860

[26] Chaumeil P-A, Mussig AJ, Hugenholtz P, Parks DH. GTDB-Tk: a toolkit to classify genomes with the Genome Taxonomy Database. Bioinformatics. 2020;36(6):1925–1927. 10.1093/bioinformatics/btz848 PMC7703759 31730192

[27] van der Walt AJ, van Goethem MW, Ramond J-B, Makhalanyane TP, Reva O, Cowan DA. Assembling metagenomes, one community at a time. BMC Genomics. 2017;18(1):521. doi: 10.1186/s12864-017-3918-9 28693474 PMC5502489

[28] Langmead B, Nellore A. Cloud computing for genomic data analysis and collaboration. Nat Rev Genet. 2018;19(4):208–219. doi: 10.1038/nrg.2017.113 29379135 PMC6452449

[29] Bharti R, Grimm DG. Current challenges and best-practice protocols for microbiome analysis. Brief Bioinformatics. 2021;22(1):178–193. doi: 10.1093/bib/bbz155 31848574 PMC7820839

[30] Meyer F, Fritz A, Deng Z-L, Koslicki D, Lesker TR, Gurevich A, et al. Critical Assessment of Metagenome Interpretation: the second round of challenges. Nat Methods. 2022;19(4):429–440. doi: 10.1038/s41592-022-01431-4 35396482 PMC9007738

[31] Bagheri H, Severin AJ, Rajan H. Detecting and correcting misclassified sequences in the large-scale public databases. Bioinformatics. 2020;36(18):4699–4705. doi: 10.1093/bioinformatics/btaa586 32579213 PMC7821992

[32] Loeffler C, Karlsberg A, Martin LS, Eskin E, Koslicki D, Mangul S. Improving the usability and comprehensiveness of microbial databases. BMC Biol. 2020;18(1):37. doi: 10.1186/s12915-020-0756-z 32264902 PMC7140547

[33] Vuong P, Lim DJ, Murphy DV, Wise MJ, Whiteley AS, Kaur P. Developing Bioprospecting Strategies for Bioplastics Through the Large-Scale Mining of Microbial Genomes. Front Microbiol. 2021;12(1950). doi: 10.3389/fmicb.2021.697309 34322108 PMC8312272

[34] Georgeson P, Syme A, Sloggett C, Chung J, Dashnow H, Milton M, et al. Bionitio: demonstrating and facilitating best practices for bioinformatics command-line software. GigaScience. 2019;8(9):giz109. doi: 10.1093/gigascience/giz109 31544213 PMC6755254

[35] Wickham H, Averick M, Bryan J, Chang W, McGowan L, François R, et al. Welcome to the Tidyverse. J Open Source Softw. 2019;4:1686.

